# Classifying Genetic Lines in Pork Production by Ileal Crude Protein and Amino Acid Digestibility in Growing Pigs

**DOI:** 10.3390/ani13121898

**Published:** 2023-06-06

**Authors:** János Tenke, Orsolya Vida, István Nagy, János Tossenberger

**Affiliations:** 1 Albert Kázmér Faculty of Mosonmagyaróvár, Széchenyi István University, Lucsony Str. 2, H-9200 Mosonmagyaróvár, Hungary; tossenberger.janos@sze.hu; 2 Bonafarm Agriculture-Swine Department, Ady Endre Str. 21, H-7754 Bóly, Hungary; 3 DSM Nutritional Products Hungary Ltd., Japán Fasor 4, H-2367 Újhartyán, Hungary; orsolya.vida@dsm.com; 4 Institute of Animal Science, Hungarian University of Agriculture and Life Sciences, Kaposvár Campus, Guba Sándor Str. 40, H-7400 Kaposvár, Hungary; nagy.istvan.prof@uni-mate.hu

**Keywords:** pigs, feeding, genetic lines, growth performance, ileal digestibility, crude protein, lysine-to-energy ratio, efficiency, classification

## Abstract

**Simple Summary:**

Breeding programs in pigs have been very successful in recent decades, although the different selection directions have resulted in highly variable growth rates between the genotypes. Simultaneously, the absorption of nutrients may also show differences subject to the genotype of the animals. As far as the development of the feeding strategies is concerned, the main objective is to maximize profitability; therefore, future methods in swine nutrition must also support the different growth rates of the animals through the improved absorption of nutrients. One of the most common tools used to reduce feed costs is the optimization of the dietary lysine-to-energy ratio, because lysine is the first limiting amino acid for swine, and energy content is one of the most defining parameters of their diet. Pigs of different genetic potential can be classified with a high accuracy in respect of their crude protein and amino acid digestive capacity. Consequently, the ileal digestibility of crude protein and amino acids in growing pigs of different genotypes should serve as a basis for the development of genotype-specific feeding strategies. Further studies are needed to identify the differences in the crude protein and amino acid digestibility of cross-bred pigs with different genetic potentials.

**Abstract:**

The first aim of the study was to evaluate the effect of different dietary lysine (LYS) to energy (DE) ratios on the apparent ileal digestibility (AID) of crude protein (CP) and selected amino acids (AA) in growing pigs (40–60 kg) of different genotypes. The second aim was to classify genotypes into groups based on the AID of CP and AAs. The trials were conducted on a total of 90 cross-bred barrows (30 animals/genotype) in two replicates. Before the trial series, the experimental animals (average initial body weight (BW) = 40.9 ± 8.5 kg) were surgically fitted with post valve T-cannula (PVTC). The diets were formulated with six different total LYS/DE ratios. Titanium dioxide (TiO_2_) was added to the diets (5 g/kg) as an indigestible marker. Based on our results, it can be concluded that the LYS/DE ratio of the diets affected the AID of the CP and AA in different ways by each genotype (*p* < 0.05). It can also be concluded that pigs of different genetic potential can be classified with a high accuracy (91.7%) in respect of their CP and AA digestive capacity. Our results indicate the development of genetic-profile-based swine nutrition technologies as a future direction.

## 1. Introduction

The genetic potential and performance of pigs used in industrial pork production have gone through a substantial transformation in recent decades. The genetic lines providing the basis for pig production programs have developed into specialized sire and dam lines [1,2,3,4], which has subsequently resulted in different nutrient requirements. Differences among genotypes are also increasing due to the fact that pig breeding companies often do not publish details of their selection indices and attribute differing priorities to the various selection parameters. In addition, the application of several genome selection methods, such as Genome-Wide Association Studies (GWAS), the H matrix and Marker-assisted selection (MAS) has become quite widespread, opening new opportunities for the genetic selection of pigs [5], improving the efficiency of breeding efforts [6].

Close [7] set up three groups of pigs based on their genetic potential, namely animals with traditional, medium and high genetic potential. These categories were based on the differences among the average daily gain (ADG) and the protein content of the empty body (i.e., the sum weight of carcass, the empty gastrointestinal tract (GIT) and other non-carcass components). Already in the late 1980’s, these genotypes used in pork production were also categorized based on the average daily feed intake (ADFI), feed conversion rate (FCR), backfat thickness (BFT) and protein deposition (PD) of the animals [2], and differences determined in their growth intensity have become accepted as well [8].

The literature data also indicate that in addition to the differences among dietary performance parameters and the PD levels of the animals, the digestive efficiencies of the various pig genotypes also differ, especially when fed with high-crude-fiber diets [9,10,11]. This fact can be explained by the weight and length of the GIT as well. Specifically, research reports have identified several quantitative trait loci (QTL) in the pig genome, influencing the weight of the small and large intestine of growing and finishing pigs [12]. It is also currently known that the length of the small intestine is affected by several QTL as well [13,14,15]. A longer small intestine is more advantageous because higher levels of the dietary N compounds (peptides and AAs) can be absorbed through its larger surface from the chyme passing through at a standard transit time, and it can be assumed on this basis that the CP and AA digestive performance of the different pig genotypes can show measurable differences as well.

The ideal protein concept provides the basis for practical AA supply, and it was Mitchell [16] who first suggested its use almost 60 years ago. Concepts based on total AA requirements were developed in the early 1980’s [17,18,19]; however, it was Chung and Baker [20] who developed the practical application of this theory a decade later. Now, the diets of pigs raised in intensive production systems are generally formulated according to the ideal protein concept, based on the LYS levels in the feed. This follows from the fact that for pigs, LYS is the first limiting AA [21]. Ideal protein concepts determined on a digestible-AA basis [22] take into account the fact that the protein synthesis of pigs use only those AAs that are absorbed up to the terminal ileum [23]. Digestibility, however, may vary by genotype, although there are insufficient and often contradictory data available in this regard [8,9,10,24].

With respect to the above, our research objective was to determine how CP and AA digestibilities varied when diets formulated on the basis of the ideal protein concept but having different LYS levels were fed, and to define the characteristics of growing pigs of different genotypes based on these variances.

## 2. Materials and Methods

### 2.1. Animals and Facilities

The trials were conducted with cross-bred growing pigs of 3 different genotypes from different lines. The genotypes investigated during the trial are shown in Table 1. Animals were free from brucellosis, leptospirosis, Aujeszky’s disease, Porcine Reproductive and Respiratory Syndrome (PRRS) and *Brachyspira hyodysenteriae*. The effect of 6 different total LYS/DE ratios were evaluated. Ten animals per treatment per genotype received the diets of 40.0–60.0 kg live weight in two replicates (*n* = 10/treatment/genotype). The genotype and the performance traits of the experimental animals investigated during the trial are shown in Table 1.

Before the trials, when the barrows reached approximately 28.0 kg live weight, all of the animals (30 animals/genotype) were surgically fitted with medical silicone PVTC-cannulas of 25 mm internal diameter, according to the method described by Van Leeuwen et al. [25]. The trials were conducted at Kaposvár University, Department of Animal Nutrition, in accordance with the declaration of the Hungarian National Scientific Ethical Committee of Animal Experimentation for studies involving animals (approval number: SOI/31/446-7/2014.).

The average initial bodyweight BW of the animals was 40.9 ± 8.5 kg. Before the surgeries, during the regeneration and adaptation period and under the collecting period the pigs were housed individually in solid-floor, smooth-plastic-walled pens (1.8 × 1.4 m/2.5 m^2^/animal), in a climate-controlled barn with an air temperature of 19–22 °C. A trough feeder and a stainless-steel nipple drinker were installed for each pen. Particular attention was paid to keeping the pigs washed, and skin irritation was minimized by the application of a zinc oxide spray. From 0700 to 1900 h the experimental room was artificially illuminated; during the night the lights were dimmed. The animals could move freely in the pens.

### 2.2. Dietary Treatments

The experimental diets were formulated on corn, barley and soybean meal (SBM) base, according to the recommendation of the NRC (1998) [26], taking into consideration the ideal protein concept (Table 2 and Table 3). The experimental diets were formulated with a constant DE, CP and crude-fiber (CF) content but with 6 different total LYS levels (LYS/DE ratio: A: 0.55; B: 0.61; C: 0.68; D: 0.74 E: 0.80; F: 0.82).

The DE value of each diet was calculated using the equation described by Noblet and Perez [27] as follows: DE (kcal/kg) = 4168 − (9.1 × %Ash) + (19 × %CP) + (39 × %Ether Extract) − (36 × %Neutral Detergent Fiber).

In all diets, vitamin and mineral premix was also supplemented to meet or exceed the nutrient requirement estimates suggested by the NRC (1998) [26]. Titanium dioxide (TiO_2_/Huntsman Tioxide^®^ A-HR, Sachtleben Chemie GmbH., Dr. Rudolf-Sachtleben Str. 4, Duisburg, Germany) was added on top (5 g/kg) of the diets as an indigestible marker. Pigs were fed twice daily (at 0830 and at 1630 h) in two equal portions at a level of 2.8 times their maintenance energy requirement (450 kJ of ME/kg of BW^0.75^). The physical form of the experimental diets was finely ground meal feed. Water was mixed with the feed (2.5:1) just before feeding. Animals had free access to drinking water during the experiment.

### 2.3. Data and Sample Collection

The digestibility studies started with a 5 d operation series. Following the surgery, the pigs were returned to the floor pens and allowed a recovery period of minimum 10 d. The recovery period was followed by a 5 d adaptation and a 3 d collection period *(*Table 4). At the beginning and at the end of each period, the BW of the pigs was individually recorded and the feed consumption for each animal was adjusted.

Between the collection days we provided rest days for the animals (Table 4). Ileal digesta samples were collected between 0830 and 1630 h on collecting days. During sample collection, cannulas were opened and a polyethylene plastic bag was attached to the cannula, permitting the flow of the digesta into the bag. The attached bags were inspected every 30 min and the filled bags were replaced with a new bag. After removing the full bags, the digesta samples were weighed and stored at −20 °C to prevent bacterial degradation of AAs. At the end of experiment, the ileal digesta samples from each pig were pooled, subsampled, lyophilized and ground before the laboratory analyses.

### 2.4. Laboratory Analysis

Based on the methods set forth in AOAC International [28], samples from feed ingredients, trial diets, and ileal digesta were analyzed for dry matter (DM) (method 930.15) and CP (method 990.03); AA concentrations, except for sulfur-containing AAs, were also analyzed by hydrolyzing with 6 N HCl for 24 h at 110 °C (method 994.13). Methionine and cystine were determined by hydrolyzation with 7.5 N HCl following a cold performic acid oxidation overnight (method 994.12). Individual AAs were measured by using an AAA 400 Ingos automatic AA analyzer (Ingos Corp., Prague, Czech Republic). The concentration of AAs was calculated on an as-fed basis (g AA/kg feed), except for tryptophan. The experimental diets were analyzed for ether extract (method 920.39), crude fiber (method 978.10), ash (method 942.05), Ca (method 978.02), and P (method 946.06). Concentrations of TiO_2_ in the diets and ileal digesta samples were analyzed as described by Myers et al. [29], using a Secomam Anthelie UV/Visible light spectrophotometer (Secomam, Alès, France).

### 2.5. Calculations

The AID of CP and AA were calculated based on CP, AA and TiO_2_ concentrations in experimental diets and ileal digesta. The AID of CP or AA were calculated based on the following equation [30]:

AID (%) = [1 − (TiO_2input_/TiO_2output_) × (AA_output_/AA_input_)] × 100

where AID is apparent ileal digestibility of CP or AA (%); TiO_2input_ and TiO_2output_ represent the TiO_2_ concentration (g/kg DM) in the diets and ileal digesta samples, respectively; and AA_input_ and AA_output_ represent the CP or AA concentrations (g/kg DM) in the diets and ileal digesta samples, respectively.

### 2.6. Statistical Analyses

Statistical analyses of the trial series were carried out with SAS/STAT^®^ 9.4 [31]. A 2-factor ANOVA model was used to determine the effects of the treatments on the AID of CP and selected AAs. The equation for the ileal digestibility traits was:

Y_ijk_ = μ + A_i_ + B_j_ + (A × B)_ij_ + e_ijk_

where: Y_ijk_ = measurements; μ = overall mean; A_i_ = main effect of treatment level (i = 6 levels); B_j_ = main effect of replicates (j = 2 replicates); (A × B) = interaction effect of treatment and replicates; and e_ijk_ = residual error. For the 2-factor ANOVA, the PROC MIXED routine was used. As for statistical reliability, the differences were verified by the Tukey test. The relationship between the treatments and the AID of the CP and selected AAs was also tested by nonlinear regression analysis (NLIN procedure).

Analysis of the AID of CP and the selected AAs consisted of a stepwise discriminant procedure and discriminant analysis, respectively, applying the STEPDISC and DISCRIM procedures, where the genetic potential of the animals served as the class variable. The equality of variance-covariance matrices of the different genotypes and the equality of their vector means were tested with the POOL and MANOVA options of the DISCRIM procedure. To ensure the reliability of the calculated discrimination function, the classification of the analyzed genotypes was tested using cross-validation.

## 3. Results

### 3.1. Ileal Digestibility of CP

**Figure 1:**
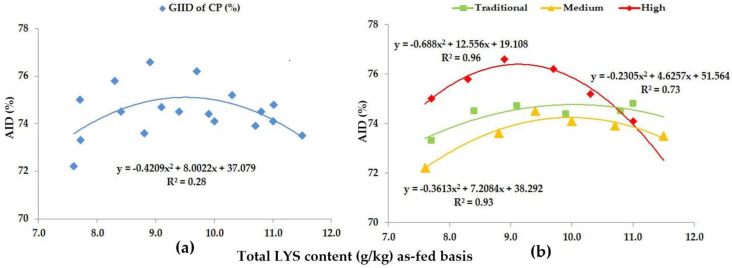
The AID (%) of CP: (**a**) The GIID evaluation. (**b**) The GSID evaluation.

The digestibility of the CP and AA content of the diets were evaluated independently of the genotype (Genotype-independent ileal digestibility—GIID) and from a genotype-specific aspect (Genotype-specific ileal digestibility—GSID). Table 5 shows the changes of the ileal GSID of CP as a function of dietary LYS/DE ratio for growing pigs with different genetic potentials, while the regression analysis of the GIID and GSID of CP is shown in Figure 1.

Our data suggest a weak relationship (R^2^ = 0.28) between the GIID of CP and the dietary LYS/DE ratio (Figure 1a) with the GSID evaluation method (Figure 1b); however, the relation was found to be strong (R^2^ = 0.73–0.96). In pigs with traditional genetic potential the digestibility of CP only showed significant growth (*p* < 0.05) as a result of the highest dietary LYS/DE ratio (Treatment F, LYS/DE: 0.82). In contrast, pigs with medium genetic potential showed improved digestibility already as a result of Treatment C (LYS/DE: 0.68) and this improvement was statistically verifiable (*p* < 0.05). The further treatments (Treatments D-, E- and F; LYS/DE: 0.74, 0.80, 0.82) did not yield any further significant improvement in CP digestibility in this genotype.

In pigs with a high genetic potential, the digestibility of CP again showed a statistically verifiable improvement with Treatment C (LYS/DE: 0.68) (*p* < 0.05); the further treatments (Treatments D-, E- and F; LYS/DE: 0.74, 0.80, 0.82) led to a gradual decline in CP digestibility (*p* < 0.05). Our data suggest that in pigs with high genetic potential, the digestibility of CP is the most efficient when feeding a diet with 0.68 LYS/DE (Treatment C), and this was measured with a 9.4 g/kg total dietary LYS content.

### 3.2. Ileal Digestibility of AAs

**Figure 2:**
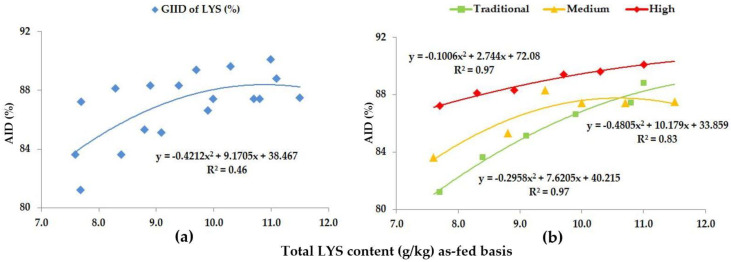
The AID (%) of LYS: (**a**) The GIID evaluation. (**b**) The GSID evaluation.

**Figure 3:**
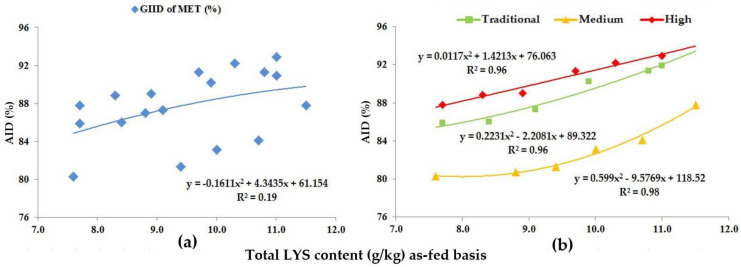
The AID (%) of MET: (**a**) The GIID evaluation. (**b**) The GSID evaluation.

**Figure 4:**
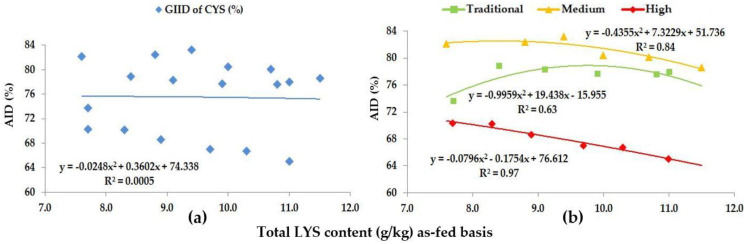
The AID (%) of CYS: (**a**) The GIID evaluation. (**b**) The GSID evaluation.

**Figure 5:**
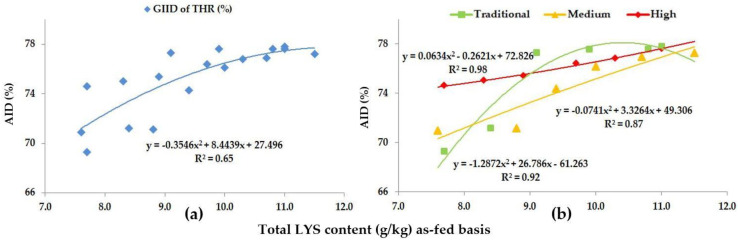
The AID (%) of THR: (**a**) The GIID evaluation. (**b**) The GSID evaluation.

**Figure 6:**
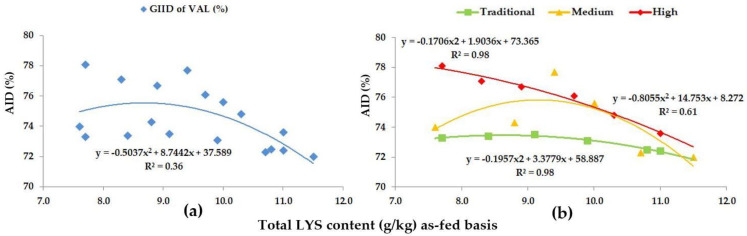
The AID (%) of VAL: (**a**) The GIID evaluation. (**b**) The GSID evaluation.

**Figure 7:**
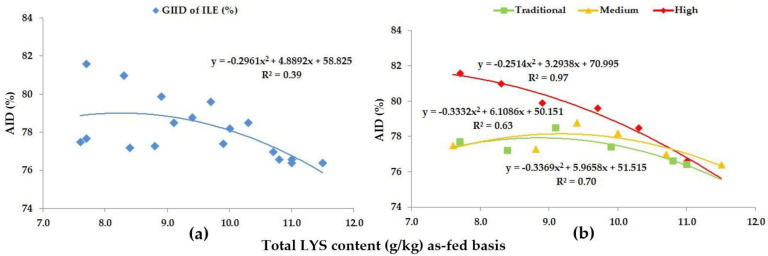
The AID (%) of ILE: (**a**) The GIID evaluation. (**b**) The GSID evaluation.

**Figure 8:**
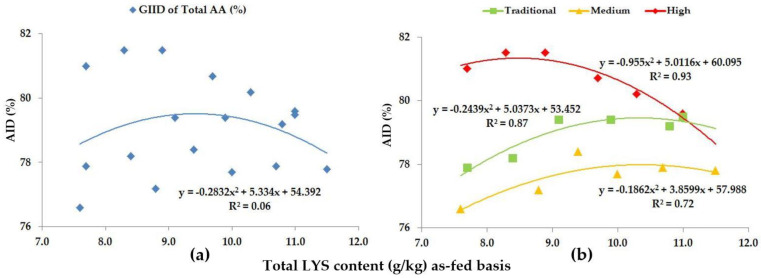
The AID (%) of Total AA: (**a**) The GIID evaluation. (**b**) The GSID evaluation.

Table 5 presents the changes in the digestibility of the studied AAs (LYS, MET, CYS, THR, VAL, ILE). The regression analyses of AA digestibility are shown in Figure 2, Figure 3, Figure 4, Figure 5, Figure 6, Figure 7 and Figure 8 as evaluated by GIID and GSID. When evaluating the digestibility of LYS irrespectively of the genotype (Figure 2a), a moderate relationship (R^2^ = 0.46) was found between the dietary LYS/DE ratio and the digestibility of LYS.

The genotype specific evaluation (GSID), however, shows a more accurate picture of the changes in digestibility (Figure 2b), because in this case, the relation between dietary LYS/DE ratios and the digestibility of LYS is strong (R^2^ = 0.83–0.97). The digestibility of LYS improved significantly with the increase in the LYS/DE ratios of the experimental diets (*p* < 0.05), but this improvement in digestibility was different for the different genotypes (Table 5); furthermore, within the different genotypes the digestibility was the highest when feeding diets with different dietary LYS/DE ratios. The digestibility of LYS moved between 81.2% (Treatment A; LYS/DE: 0.55) and 90.1% (Treatment F; LYS/DE: 0.82). The digestibility of LYS shows a relative 10.0% difference depending on the LYS/DE ratio of the diets and the genetic potential of the animals.

Based on the GIID evaluation, the relation between the digestibility of MET and the LYS/DE ratio (Figure 3a) is weak (R^2^ = 0.19). When applying the GSID evaluation (Figure 3b), however, there is a strong relationship between both the dietary LYS/DE ratio and the digestibility of MET in all studied genotypes (R^2^ = 0.96–0.98).

Based on the GIID evaluation (Figure 4a) the relationship is the weakest between the digestibility of CYS and the LYS/DE ratio (R^2^ = 0.0005). Taking the GSID evaluation (Figure 4b) as a basis, however, there is a strong relationship between the LYS/DE ratio and the digestibility of CYS (R^2^ = 0.63–0.97). Digestibility of CYS was found to be between 65.0% (Treatment F; LYS/DE: 0.82) and 81.2% (Treatment A; LYS/DE: 0.55).

Based on the GSID analysis, the digestibility of CYS showed a difference of in excess of 17.0% subject to the LYS/DE ratio of the diet and the genetic potential of the animals.

As for THR, the GIID evaluation method (Figure 5a) found a strong relationship between the LYS/DE ratio and the digestibility of THR (R^2^ = 0.65); on the basis of the GSID evaluation (Figure 5b), however, the relationship between the LYS/DE ratio and the digestibility of THR was found to be even stronger (R^2^ = 0.87–0.98).

The digestibility of THR moved between 69.3% (Treatment A; LYS/DE: 0.55) and 77.8% (Treatment F; LYS/DE: 0.82), reflecting an 8.5% improvement in the digestibility.

In reference to VAL, the GIID evaluation method (Figure 6a) showed a weak relationship between the LYS/DE ratio and the digestibility of VAL (R^2^ = 0.36). Based on the GSID evaluation (Figure 6b), however, there is a strong relation between the LYS/DE ratio and the digestibility of VAL (R^2^ = 0.61–0.98). The digestibility of VAL shows a 6.1% difference, depending on the LYS/DE ratio of the diets and the genetic potential of the animals.

As for ILE, based on the GIID (Figure 7a), the relationship between the LYS/DE ratio and the digestibility of ILE is R^2^ = 0.39. The relation between the LYS/DE ratio and the digestibility of ILE based on the GSID evaluation (Figure 7b) is stronger in this case as well (R^2^ = 0.63–0.97). The data in Figure 7 and Table 5 show that the difference in the digestibility of ILE can be up to 9.1% subject to the LYS/DE ratio of the diets.

When looking at the Total AA levels of the diets, we included the digestibility of all AAs (LYS, MET, CYS, THR, VAL, ILE, LEU, HIS, PHE, TYR, ARG, ASP, GLU, ALA, SER, GLY, PRO) except for TRP. The GIID evaluation method (Figure 8a) is not suitable for describing the relationship between the digestibility of the total AA content, and the LYS/DE ratio of the diets (R^2^ = 0.06). Based on the GSID evaluation method, however, the relationship between the LYS/DE ratio and the digestibility of total AA group is strong (R^2^ = 0.72–0.93).

### 3.3. Classification Results

Our preliminary hypothesis proposed that trial animals can be classified into predetermined groups based on the different CP and AA digestive capacity of growing pigs of different genotypes and genetic potentials. The GSID evaluation method presented in Figure 1, Figure 2, Figure 3, Figure 4, Figure 5, Figure 6, Figure 7 and Figure 8 well illustrates these differences.

Taking into account the number of experimental animals excluded from the trials (n = 19), the total evaluable data set was n = 161. We conducted a descriptive statistical analysis (Table 6) to arrange the trial animals (n = 161) into groups in order to compare the treatment independent average of CP and AAs ileal digestibilities.

We followed the descriptive statistical analysis with a stepwise discriminant analysis using the STEPDISC procedure in order to select those of the studied digestibility parameters that were the most suitable for the purpose of classifying the genotypes. We used the FORWARD option for this purpose and the PROC STEPDISC in each case produced the variable which improved the discriminative ability of the model. The results of the procedure are shown in Table 7.

Based on the discriminant analysis, the variance-covariance matrices (Chi^2^ = 0.001) and the mean vectors (Wilks lambda = 0.001) were different; therefore, we applied a quadratic discriminant analysis. The classification of the calibration data based on the discriminant function with and without cross validation is shown in Table 8.

Using the cross-validated quadratic discriminant function, 87.5% of the animals having traditional genetic potential could be accurately assigned to their own genotype. For animals of a medium genetic potential, the cross-validation method yielded accurate classification in 92.0% of the cases, and for animals with a high genetic potential the accuracy of classification was 96.4%. Based on the results of the cross-validation procedure, the average accuracy of classification was 91.9% irrespective of the genotype.

## 4. Discussion

There is only limited and often contradictory literature data available about the relationship between the digestibility of nutrients and the genotype of the animals [8,9,10,24]; nevertheless, our data can be compared to the findings of a few researchers regarding the digestibility of CP. In our studies, the digestibility of CP was 74.5% and 74.7%, respectively, when Treatment C (CP: 155.0 g/kg; Total LYS: 9.4 g/kg) was fed to animals of traditional ([Yorksire × Landrace] × Duroc) and medium ([Hungarian Yorkshire × Hungarian Landrace] × [Hampshire × Pietrain]) genetic potential. Adebiyi et al. [32] found a 74.0% CP digestibility in (Yorkshire × Landrace) cross-bred pigs when feeding them a corn-soy based trial diet (CP: 155.0 g/kg; Total LYS: 9.0 g/kg). She et al. [33], whilst also feeding a corn-soy based diet (CP: 179.0 g/kg; Total LYS: 11.0 g/kg), found a 77.4% CP digestibility in PIC L 359 × Camborough cross-bred pigs. This result is the nearest to the 76.6% CP digestibility measured in our studies, when we fed Treatment C (CP: 155.0 g/kg; Total LYS: 9.4 g/kg) to animals of a high genetic potential ([Danish Yorkshire × Danish Landrace] × Danish Duroc). Yoo et al. [34], in contrast, stated a higher CP digestibility in ([Yorksire × Landrace] × Duroc) cross-bred animals. According to the data of the authors, CP digestibility was 85.0% when a corn-fishmeal-based diet, with 155.0 g/kg CP and 9.8 g/kg Total LYS content, was fed to pigs. In our studies, we found CP digestibility to be the highest in the animals with the highest genetic potential, but this remained 8.4% below the value measured by Yoo et al. [33] (76.6%). Although the digestibility of CP differs in the studies referenced, it cannot be stated clearly on this basis that the genotype of the animals should be listed among the factors affecting the digestibility of CP (differences between the experimental methods, housing conditions, health status of the animals, feed ingredients and diets, BW, etc.).

Trial data, however, confirm our preliminary hypothesis, that the digestibility of CP and of the studied AAs differ subject to the genetic potential/genotype of the trial animals as well. Based on the data the animals of traditional, medium and high genetic potential can be classified at a high accuracy (91.9%), using discriminant analysis (Table 8). The difference of efficiency of CP and AA digestion in the studied genotypes can be deemed as proven based on the characterization of these differences in digestion physiology. According to our data, the use of the GSID evaluation method can be of huge importance because the digestibility of CP and AAs is affected by the LYS/DE ratio of the diets in a different way in animals with a different genetic potential. The GIID evaluation method does not reflect these differences, as it underestimates the efficiency of absorption in some cases and overestimates the same in others. It can be declared that the studied growing pigs of different genetic potentials cannot be managed uniformly because their digestion efficiency of CP and AAs is different. The results of our studies highlight that the ileal digestible portion of CP and AAs (that can potentially be utilized for protein biosynthesis) changes with the genotype/genetic potential of the animals. This allows us to draw the conclusion that the differences found in the digestibility of CP and AAs may exist not only in the case of compound diets but also in the case of feed ingredients. The studies aiming to determine the digestible AA content of feed ingredients, however, do not include the standardization of the genotype of the animals that is the genotype specific digestibility coefficients are not generally used. Our findings, however, clearly point out the future necessity of their application. In addition to the different absorption efficiency of CP and AAs by genotypes it should also be noted that the peak of digestibility can be achieved by feeding diets with different LYS/DE levels (LYS content) to the different genotypes, which should also be considered when providing the AA requirement of the animals. Based on these differences, the accuracy of AA requirements of the animals can and should be further refined in the future. It also appears necessary, furthermore, to conduct studies to clarify the background of higher CP and AA digestibility in other high-genetic-potential-animals, with special regard to traits referred in the literature [11,12]: the length, mass and maturity of the GIT of the animals.

The results indicate as a future direction the development of data bases using genotype-specific ileal AA digestibilities, and the widespread practical application of genetic-profile-based swine nutrition technologies.

## 5. Conclusions

The results suggest that there are statistically verifiable differences in the CP and AA absorption of cross-bred pigs with different genetic potentials used in intensive pork production. Our data highlight that the AA requirements formulated on ileal digestible AA basis should include the use of digestibility coefficients developed through the genotype specific evaluation method. In the absence of this approach, the real AA requirement of the animals may be distorted by inaccuracies. It is suggested that the GSID of CP and amino AAs be taken into account, which can contribute to the more accurate supply of the AA requirement of growing pigs and consequently to the more optimal biological, economic and ecologic efficiency of pork production.

## Figures and Tables

**Table 1:** The classification of the experimental animals based on the performance traits of the breeds.

Performance Traits	Genetic Potential		
	Traditional ^1^	Medium ^1^	High ^1^
	[Hungarian Yorkshire × Hungarian Landrace] × Duroc	[Hungarian Yorkshire × Hungarian Landrace] × [Hampshire × Pietrain]	[Danish Yorkshire × Danish Landrace] × Danish Duroc
Initial BW (kg)	27.0	25.4	24.0
Slaughter weight (kg)	119.6	119.3	118.1
ADFI (g/day)	2396	2517	2789
ADG (g/day)	765	903	1046
FCR (kg/kg)	2.92	2.75	2.66
Fattening phase (day)	121	104	90

^1^ Groups were classified by Close [7].

**Table 2:** Composition and nutrient content of the experimental diets (g/kg as-fed basis).

Ingredients	Treatment					
	A	B	C	D	E	F
Corn	503.0	494.7	505.0	531.0	536.0	549.0
Barley	251.0	274.0	265.0	240.0	237.0	225.0
Extr. SBM	191.8	187.0	183.6	179.6	175.0	171.5
Arbocel ^1^	11.0	8.0	8.0	8.0	8.0	8.0
Sunflower oil	15.0	7.0	7.4	7.5	7.5	7.6
MCP	7.5	7.5	7.5	7.6	7.6	7.7
Limestone	10.0	10.0	10.0	10.0	9.9	9.9
NaCl	4.1	4.1	4.2	4.2	4.2	4.2
Premix (0.5%) ^2^	5.0	5.0	5.0	5.0	5.0	5.0
Lysine-HCl 78% ^3^	1.0	1.8	2.9	4.0	5.1	6.2
DL-methionine 99% ^3^	0.0	0.1	0.6	0.9	1.2	1.5
L-cystine-HCl 98% ^3^	0.0	0.0	0.0	0.3	0.6	0.9
L-threonine 98% ^3^	0.0	0.0	0.5	1.1	1.7	2.2
L-tryptophan 98% ^3^	0.0	0.0	0.0	0.2	0.4	0.6
Total	1000.0	1000.0	1000.0	1000.0	1000.0	1000.0
Nutrient content						
DEs ^4^ (MJ/kg)	14.0	13.8	13.8	13.8	13.8	13.8
Dry matter ^5^	904.0	900.3	903.0	900.0	902.7	902.3
CP ^5^	153.7	153.7	155.0	155.0	155.0	155.3
Ether extract ^5^	37.7	30.0	30.3	30.3	30.3	30.3
Crude fiber ^5^	46.7	45.0	45.0	45.0	45.7	44.0
LYS_total_^5^	7.7	8.4	9.4	10.2	11.0	11.3
Ca ^5^	5.6	5.2	5.4	5.3	5.3	5.4
P ^5^	5.0	4.9	5.1	5.0	5.1	5.1
Total LYS/DE ratio ^4^	0.55	0.61	0.68	0.74	0.80	0.82

^1^ Produced by J. Rettenmaier & Söhne GmbH. ^2^ Provided the following per kilogram of premix: Vitamin A: 1,750,000 IU, Vitamin D3: 350,000 IU, Vitamin E: 8750 mg, Vitamin K3: 350 mg, Vitamin B1: 262.5 mg, Vitamin B2: 875 mg, Vitamin B3: 2100 mg, Vitamin B6: 700 mg, Vitamin B12: 4375 mg, Biotin: 21 mg, Folic acid: 105.07 mg, Choline: 24,000 mg, Fe: 19,175 mg, Zn: 20,001 mg, Mn: 6488.3 mg, Cu: 2225 mg, Co: 6.5 mg, I: 65 mg, Se: 67.75 mg. ^3^ Produced by Ajinomoto. ^4^ Calculated value. ^5^ Analyzed chemical composition.

**Table 3:** Analyzed AA composition of the experimental diets (g/kg as-fed basis).

Amino Acids	Treatment					
	A	B	C	D	E	F
Essential amino acids ^1^						
Lysine	7.7	8.4	9.4	10.2	11.0	11.3
Methionine	2.4	2.5	2.8	3.0	3.6	3.9
Cystine ^2^	2.3	2.3	2.2	2.3	2.2	2.2
Threonine	5.8	5.8	6.4	6.7	7.0	7.5
Valine	7.3	7.1	7.2	7.1	6.9	7.0
Leucine	13.2	13.3	13.3	13.3	13.1	13.0
Isoleucine	6.1	6.0	6.0	6.0	5.8	5.8
Histidine	4.2	4.1	4.3	4.2	4.3	4.3
Phenylalanine	7.7	7.9	7.8	7.7	7.5	7.6
Tyrosine ^2^	4.2	4.4	4.4	4.3	4.3	4.2
Non-essential amino acids						
Arginine	8.7	8.5	8.5	8.3	8.3	8.0
Aspartic acid	3.4	14.2	14.2	13.9	13.7	13.7
Glutamic acid	31.0	30.5	29.8	29.4	29.7	29.0
Alanine	8.3	8.3	8.3	8.3	8.1	8.1
Serine	7.7	7.8	7.7	7.7	7.6	7.5
Glycine	6.5	6.4	6.4	6.3	6.1	6.1
Proline	11.5	11.5	11.5	11.5	11.2	11.4

^1^ Except tryptophan. ^2^ Semi-essential amino acid.

**Table 4:** Timeline of the digestibility studies.

Preparation and Digesta Collecting							
Implantation of PVTC cannulas 5-d	Regeneration period Min. 10-d	Adaptation period 5-d	Collecting Period				
			Collecting day	Rest day	Collecting day	Rest day	Collecting day

**Table 5:** The GSID of CP and AA in growing pigs with different genetic potentials.

Genotype Specific Ileal Digestibility (%)							
Nutrient	Treatment ^1,2^						
	A	B	C	D	E	F	RMSE
Traditional							
CP	73.3 ^a^	74.5 ^ab^	74.7 ^ab^	74.4 ^ab^	74.5 ^ab^	74.8 ^b^	1.5
Lysine	81.2 ^a^	83.6 ^b^	85.1 ^c^	86.6 ^d^	87.4 ^d^	88.8 ^e^	1.0
Methionine	85.9 ^a^	86.0 ^a^	87.3 ^b^	90.2 ^c^	91.3 ^cd^	91.9 ^d^	1.3
Cystine	73.7 ^a^	78.9 ^b^	78.3 ^b^	77.7 ^b^	77.6 ^b^	78.0 ^b^	2.8
Threonine	69.3 ^a^	71.2 ^b^	77.3 ^c^	77.6 ^c^	77.6 ^c^	77.8 ^c^	1.9
Valine	73.3	73.4	73.5	73.1	72.5	72.4	1.6
Isoleucine	77.7	77.2	78.5	77.4	76.6	76.4	1.6
TOTAL AA ^3^	77.9 ^a^	78.2 ^a^	79.4 ^b^	79.4 ^b^	79.2 ^b^	79.5 ^b^	1.0
Medium							
CP	72.2 ^a^	73.6 ^ab^	74.5 ^b^	74.1 ^b^	73.9 ^b^	73.5 ^ab^	1.4
Lysine	83.6 ^a^	85.3 ^b^	88.3 ^c^	87.4 ^c^	87.4 ^c^	87.5 ^c^	1.4
Methionine	80.3 ^a^	80.7 ^ab^	81.3 ^ab^	83.1 ^bc^	84.1 ^c^	87.8 ^d^	2.5
Cystine	82.1 ^abc^	82.4 ^ab^	83.2 ^b^	80.4 ^acd^	80.1 ^cd^	78.6 ^d^	2.1
Threonine	70.9 ^a^	71.1 ^a^	74.3 ^b^	76.1 ^bc^	76.9 ^c^	77.2 ^c^	2.0
Valine	74.0 ^ab^	74.3 ^b^	77.7 ^c^	75.6 ^b^	72.3 ^ad^	72.0 ^d^	1.8
Isoleucine	77.5 ^abc^	77.3 ^abc^	78.8 ^b^	78.2 ^ab^	77.0 ^ac^	76.4 ^c^	1.5
TOTAL AA ^3^	76.6 ^a^	77.2 ^ab^	78.4 ^c^	77.7 ^abc^	77.9 ^bc^	77.8 ^bc^	1.1
High							
CP	75.0 ^ab^	75.8 ^abd^	76.6 ^d^	76.2 ^cd^	75.2 ^bc^	74.1 ^a^	1.1
Lysine	87.2 ^a^	88.1 ^b^	88.3 ^b^	89.4 ^c^	89.6 ^cd^	90.1 ^d^	0.7
Methionine	87.8 ^a^	88.8 ^b^	89.0 ^b^	91.3 ^c^	92.2 ^d^	92.9 ^e^	0.6
Cystine	70.3 ^a^	70.2 ^a^	68.6 ^ab^	67.0 ^bc^	66.7 ^cd^	65.0 ^d^	1.8
Threonine	74.6 ^a^	75.0 ^a^	75.4 ^ab^	76.4 ^bc^	76.8 ^c^	77.6 ^c^	1.3
Valine	78.1 ^a^	77.1 ^ab^	76.7 ^b^	76.1 ^b^	74.8 ^c^	73.6 ^c^	1.3
Isoleucine	81.6 ^a^	81.0 ^ab^	79.9 ^bc^	79.6 ^cd^	78.5 ^d^	76.6 ^e^	1.1
TOTAL AA ^3^	81.0 ^ab^	81.5 ^b^	81.5 ^b^	80.7 ^ac^	80.2 ^cd^	79.6 ^d^	0.7

^1^ Total LYS content (g/kg): A: 7.7; B: 8.4; C: 9.4; D: 10.2; E: 11.0; F: 11.3. ^2^ Calculated LYS/DE ratio: A: 0.55; B: 0.61; C: 0.68; D: 0.74; E: 0.80; F: 0.82. ^3^ Total AA digestibility: Except tryptophan. RMSE: Root Mean Square Error. ^a–e^: *p* < 0.05.

**Table 6:** Descriptive statistics for GSID (%) of CP and AAs.

Nutrient	Genetic Potential	Number of Observations	Mean	SD ^1^	CV (%) ^2^
CP	Traditional	56	74.4	1.57	2.5
	Medium	50	73.7	1.53	2.3
	High	55	75.6	1.35	1.8
	Total-sample	161	74.6	1.67	2.8
Lysine	Traditional	56	85.3	2.74	7.5
	Medium	50	86.7	2.16	4.7
	High	55	88.7	1.23	1.5
	Total-sample	161	86.9	2.56	6.6
Methionine	Traditional	56	88.7	2.79	7.8
	Medium	50	82.8	3.50	12.2
	High	55	90.2	1.95	3.8
	Total-sample	161	87.4	4.18	17.5
Cystine	Traditional	56	77.3	3.22	10.4
	Medium	50	81.2	2.59	6.7
	High	55	68.1	2.61	6.8
	Total-sample	161	75.4	6.18	38.2
Threonine	Traditional	56	75.0	4.03	16.3
	Medium	50	74.4	3.21	10.3
	High	55	75.9	1.69	2.8
	Total-sample	161	75.1	3.18	10.1
Valine	Traditional	56	73.1	1.65	2.7
	Medium	50	74.4	2.68	7.2
	High	55	76.2	1.94	3.8
	Total-sample	161	74.6	2.47	6.1
Isoleucine	Traditional	56	77.3	1.72	2.9
	Medium	50	77.6	1.66	2.8
	High	55	79.6	1.96	3.9
	Total-sample	161	78.2	2.06	4.2
Total AA	Traditional	56	78.9	1.17	1.4
	Medium	50	77.6	1.26	1.6
	High	55	80.8	0.96	0.9
	Total-sample	161	79.2	1.72	3.0

^1^ SD: Standard Deviation. ^2^ CV (%): Coefficient of Variation.

**Table 7:** Stepwise selection summary.

Step	Nutrient	R-Square	F Value	Pr > F
1	CP	0.22	22.3	<0.0001
2	Lysine	0.31	35.9	<0.0001
3	Methionine	0.56	100.6	<0.0001
4	Cystine	0.79	301.8	<0.0001
5	Threonine	0.04	3.1	0.0491
6	Valine	0.28	30.2	<0.0001
7	Isoleucine	0.26	27.5	<0.0001
8	Total AA	0.57	105.8	<0.0001

**Table 8:** Classification and cross-validation summary for calibration data (n = 161).

Number of Observations and Percent Classified					
From Genotype		Into Genotype			
		Traditional	Medium	High	Total
Original ^1^					
Number of observations	Traditional	54	2	0	56
	Medium	3	47	0	50
	High	1	0	54	55
%	Traditional	96.4	3.6	0.0	100.0
	Medium	6.0	94.0	0.0	100.0
	High	1.8	0.0	98.2	100.0
Cross-Validated ^2^					
Number of observations	Traditional	49	7	0	56
	Medium	4	46	0	50
	High	2	0	53	55
%	Traditional	87.5	12.5	0.0	100.0
	Medium	8.0	92.0	0.0	100.0
	High	3.6	0.0	96.4	100.0

^1^ 96.3% of original grouped cases correctly classified. ^2^ 91.9% of cross-validated grouped cases correctly classified.

## Data Availability

The data presented in this study are available on request from the corresponding author. The study did not include humans.
